# Simulation-based learning in palliative care in postgraduate nursing education: a scoping review

**DOI:** 10.1186/s12904-023-01149-w

**Published:** 2023-03-29

**Authors:** Karoline Skedsmo, Andréa Aparecida Gonçalves Nes, Hege Vistven Stenseth, Kristin Hofsø, Marie Hamilton Larsen, Deborah Hilderson, Dieter Smis, Carina Lundh Hagelin, Camilla Olaussen, Marianne Trygg Solberg, Hanne Maria Bingen, Mia Alexandra Ølnes, Simen A. Steindal

**Affiliations:** 1 Lovisenberg Diaconal University College, Lovisenberggt. 15B, 0456 Oslo, Norway; 2 Department of Otorhinolaryngology, Head and Neck Surgery, Oslo University Hospital, Oslo, Norway; 3 Department of Research and Development, Division of Emergencies and Critical Care, Oslo University Hospital, Oslo, Norway; 4 Karel de Grote University College, Antwerp, Belgium; 5 GZA Hospitals, Antwerp, Belgium; 6 Department of Health Care Sciences, Palliative Research Centre, Marie Cederschiöld University, Stockholm, Sweden; 7 Department of Neurobiology, Care Sciences and Society, Division of Nursing, Karolinska Institutet, Stockholm, Sweden; 8 Faculty of Health Studies, VID Specialized University, Oslo, Norway

**Keywords:** Education, Nursing, Palliative care, Postgraduate studies, Review, Simulation

## Abstract

**Background:**

Nurses require advanced competence in palliative care, but they face wide variations in education and a shortage in opportunities for clinical placement. Simulation-based learning (SBL) can enable students to develop clinical skills, critical thinking and confidence. No scoping reviews to date have mapped the use of SBL in palliative care within postgraduate nursing education.

**Methods:**

The aim of this scoping review was to systematically map published studies on the use of SBL in palliative care in postgraduate nursing education. A scoping review was conducted using Arksey and O’Malley’s (Int J Soc Res Meth 8(1):19–32, 2005) methodological framework. A systematic and comprehensive search of the Cumulative Index to Nursing and Allied Health Literature (CINAHL), the Education Resources Information Center (ERIC), Ovid MEDLINE, Ovid EMBASE, Allied and Complementary Medicine and PsycINFO was performed for studies published between January 2000 and April 2022. Two authors independently assessed papers for inclusion and extracted data. Reporting followed the Preferred Reporting Items for Systematic Reviews and Meta-Analyses extension for Scoping Reviews (PRISMA-ScR) checklist. The protocol was registered on the Open Science Framework.

**Results:**

This review includes 10 studies. Three thematic groupings were identified: enhanced understanding of the importance of teamwork, interdisciplinarity and interpersonal skills; preparedness and confidence in one’s ability to communicate during emotionally challenging situations; and impact and relevance to one’s own clinical practice.

**Conclusions:**

The use of SBL in palliative care in postgraduate nursing education seems to enhance students’ understanding of the importance of teamwork and interdisciplinarity. The review shows contradictory results regarding whether SBL in palliative care increases students’ confidence in their communication skills. Postgraduate nursing students experienced personal growth after participating in SBL. Because our findings indicate that limited research has been conducted within this field, future research should (1) explore postgraduate nursing students’ experiences with SBL in palliative care with a focus on more practical content such as symptom management, (2) examine the relevance and application of SBL in clinical practice, and (3) be reported in line with recommendations on the reporting of simulation research.

**Supplementary Information:**

The online version contains supplementary material available at 10.1186/s12904-023-01149-w.

## Background

The aim of palliative care is to improve the quality of life among seriously ill and dying patients and their families. A holistic approach is essential to meet the complex needs of these patients [1, 2]. Recent publications have highlighted the need for a shift from a disease-centred to a more person-centred approach in palliative care [3, 4]. Palliative care should be offered to all patients with a progressive chronic illness [5] and integrated as early as possible into the patients’ treatment trajectory [3, 6]. The number of patients requiring palliative care services due to both chronic illness and old age is expected to increase in the future [7].

Nurses belong to the largest group of healthcare professionals involved in palliative care [8]. They hold a unique position, since they are available for patients around the clock and often serve as coordinators of healthcare services [1]. Hökkä et al. [9] identified six types of competencies that nurses need in palliative care: leadership, communication, collaboration, clinical, ethical-legal, psychosocial and spiritual competence. In addition, studies have reported that nurses want more knowledge and skills in palliative care, especially regarding the palliative care philosophy and symptom management, and how to communicate with families and provide end-of-life (EOL) care [1, 10, 11]. Increased complexity in palliative care may occur when patients have multiple needs, when communication challenges arise or when the primary care staff lacks confidence [12].

Nurses’ ability to make sound clinical judgements through clinical reasoning and critical thinking is important for delivering individually tailored palliative care [13]. In contrast, inadequate nursing skills and capacities could be a potential barrier to palliative care services [14]. In order to meet the complex needs of these patients and to provide them (and their families) with high-quality palliative care, nurses require advanced education and training, but dramatic variations exist in nursing education regarding the prioritization of palliative care across Europe [8]. Further, the field of postgraduate nursing education has an evident shortage of opportunities for clinical placement [15, 16]. A solution to this challenge is necessary.

Simulation-based learning (SBL) is conceptualized in nursing education as ‘a dynamic process involving the creation of a hypothetical opportunity that incorporates an authentic representation of reality, facilitates active student engagement and integrates the complexities of practical and theoretical learning with opportunity for repetition, feedback, evaluation and reflection’, according to Bland et al., p. 668 [17]. SBL may include computer-based programmes, virtual reality, standardized patients, or manikin-based or hybrid simulations for students to learn their professional responsibilities [18, 19]. The integration of SBL can enable nursing students to develop clinical skills and increase their knowledge, critical thinking and confidence [16, 20, 21]. By using SBL based on real-life scenarios in a setting where learners feel confident to practice different skills, students may increase their critical thinking skills and practice patient-centred care and nontechnical skills without the risk of causing further strain or burden on the patients [22, 23]. Nurse practitioner students seem to be more satisfied with SBL than other learning activities [20]. The transition from traditional learning to SBL, however, may be challenging for postgraduate students, who may experience performance anxiety, be unfamiliar with simulation and perceive a lack of facilitator guidance as hinderances to their learning [24].

We conducted an initial search for previous reviews on the use of SBL in palliative care in nursing education in the databases Cinahl and Medline.

Several literature reviews have examined the research on SBL as a teaching method in palliative care. One systematic review evaluated the use of SBL as a learning experience among multidisciplinary clinical teams to learn about palliative and EOL care [25]. Other systematic reviews have examined skills training and the use of SBL to teach nursing students and healthcare professionals’ palliative care and EOL communication [18, 26]. A scoping review mapped the literature on the psychological outcomes reported following the debriefing of healthcare professionals or healthcare students who experienced expected and unexpected patient death during SBL or in clinical practice [27], while a literature review examined SBL in medical education in the training of palliative care skills. This review was not limited to nursing education, however, and the Cumulative Index to Nursing and Allied Health Literature (CINAHL) was not searched [23].

Previous literature reviews have also examined the use of SBL in palliative and EOL care simulations within undergraduate nursing education [28, 29]. Kirkpatrick et al. [29] found that in SBL involving sensitive issues that included psychosocial responses, nursing students preferred unfamiliar professional actors to portray the patient rather than high-fidelity manikins. After participating in SBL, nursing students reported several positive outcomes, including increased confidence, self-efficacy and knowledge and improved communication skills, reassurance, and understanding of the complexity and competing priorities of palliative care.

SBL is increasingly applied as a learning activity in nursing education within palliative care, and prior reviews have examined the experiences of undergraduate nursing students, but these experiences may not be transferable to SBL for postgraduate nursing students with more competence and clinical experience in palliative care. Postgraduate nursing students may also have different learning needs from undergraduate students when participating in SBL [24]. Conducting a scoping review appears relevant for summarizing the range of studies and existing findings, in addition to identifying research gaps in the research literature [30]. Our initial search for previous reviews showed that no reviews have mapped the use of SBL in palliative care in postgraduate nursing education. Therefore, the aim of this scoping review was to systematically map published studies on the use of SBL in palliative care within postgraduate nursing education.

## Methods

A scoping review was conducted using the five stages of the framework described by Arksey and O’Malley [30]: (1) identifying the research question; (2) identifying the relevant literature; (3) selecting the studies; (4) charting the data; and (5) collating, summarizing and reporting the results. The reporting of our scoping review followed the Preferred Reporting Items for Systematic Reviews and Meta-Analyses extension for Scoping Reviews (PRISMA-ScR) checklist [31]. Deviations from the published protocol (https://osf.io/agz6f/) are described in Appendix 1.

### Identifying the research question

What is known about postgraduate nursing students’ experiences in the use of SBL in palliative care?

### Identifying the relevant literature

The inclusion and exclusion criteria are shown in Table 1.

**Table 1:** Inclusion and exclusion criteria

Criteria	Inclusion	Exclusion
Participants	—Nursing students in postgraduate education, regardless of type of education and course or duration and extent of course/education —Nurses participating in simulation-based learning (SBL) in clinical practice when SLB was based on comprehensive activity where either curricula, models, textbooks or other tools for learning were developed	Preregistration nursing students or undergraduate nursing students
Phenomenon of interest	Postgraduate nursing students’ experiences of palliative care SBL in education, including manikin-based, standardized/simulated patients, computer-based programmes, virtual reality or role playing	—SBL in clinical practice not related to education —Postgraduate nursing students’ experiences in palliative care skill training
Outcomes	Nursing students reported subjective and objective outcomes	Proxy-reported outcomes (e.g. by teachers or facilitators)
Type of studies	Qualitative, quantitative and mixed-methods studies on the phenomenon, published in peer-reviewed journals	Doctoral theses, master’s theses, letters, editorials, comments, conference abstracts, books, reports or any type of review

CINAHL, the Education Resources Information Center (ERIC), Ovid MEDLINE, Ovid EMBASE, Allied and Complementary Medicine (AMED) and PsycINFO were searched to identify relevant published studies. These databases were searched from 1 January 2000 to 18 February 2021 and were updated on 21 April 2022. Because technology has improved in this time period and has played a vital role in the further development of SBL, the search was limited by year of publication. The same period has seen a strong focus on how SBL can be used to improve patient safety [22].

The search strategy was built in CINAHL by an experienced research librarian (MAØ) in collaboration with the other authors using CINAHL subject headings and text words. A second experienced research librarian peer reviewed the search strategy using the Peer Review of Electronic Search Strategies (PRESS) checklist [32]. The final CINAHL search strategy was then adapted to the subsequent databases. Detailed search histories are shown in Appendix 2.

The database search was limited to publications in Dutch, French, Portuguese, English, Spanish, Danish, Swedish and Norwegian, since the authors understand these languages and lack funding for the translation of papers. Publication-type filters were used in EMBASE and Medline to exclude letters, conference abstracts and editorials, since we only wanted to include published studies.

A hand search was performed in the reference list of papers. Studies that had cited the studies included in our review were not tracked, since the updated search should have identified these citations.

### Selecting the studies

The research librarian (MAØ) exported the search results to EndNote to remove duplicates, then exported the search results to the web application Rayyan QCRI to facilitate storage and blinding and the screening of publications for the study selection process [33].

Six pairs of authors (KS & HMB, AGGN & CO, KH & HVS, DH & DS, MHL & MTS and SAS & CLH) independently assessed whether the titles, abstracts and full-text publications met the inclusion criteria. A third author (KS, AGGN or SAS) conducted an independent assessment when there was any doubt whether a publication should be included, and discussions to reach consensus then took place. Reasons for the exclusion of full-text publications were recorded using the PRISMA 2020 flow diagram.

### Charting the data

A standardized data-charting form was developed to be able to capture relevant information on key study characteristics [31]. The content of the charting form was discussed among all the authors, and the following information was included: author, year and country; aim; participants; simulation procedures; scenarios; design; and key results related to experiences of SBL in palliative care. The charting form was piloted by the first and last authors, who extracted data from one of the articles to be included. The same six pairs of authors extracted the data. In each pair, one author extracted the data, while the other verified the data’s accuracy. Any disagreements among the two authors were resolved by an independent assessment by a third author (KS, AGGN or SAS). Agreement was based on discussion and consensus among the three authors.

### Collating, summarizing and reporting the data

In a scoping review, the results are not synthesized in the same way as in a systematic review, although some thematic construction or analytic frameworks are still warranted [30]. To answer the research question, the extracted results from the results section of the papers included in the review were thematically summarized and organized. KS read the results several times to gain an overview of the whole data material and then read the results to identify any patterns of differences and similarities across the papers regarding postgraduate nursing students’ experiences of SBL in palliative care. During this process, AAGN and SAS asked critical questions to facilitate alternative interpretations and groupings of the data [34, 35]. We used a low level of interpretation and abstraction. The findings were thoroughly discussed among all the authors, all of whom agreed upon the final thematic groupings. Trustworthiness was enhanced by the diverse research and pedagogical expertise of the authors, several of whom have extensive competence in SBL or palliative care. The discussions among the authors thus facilitated competing interpretations.

## Results

**Fig. 1:**
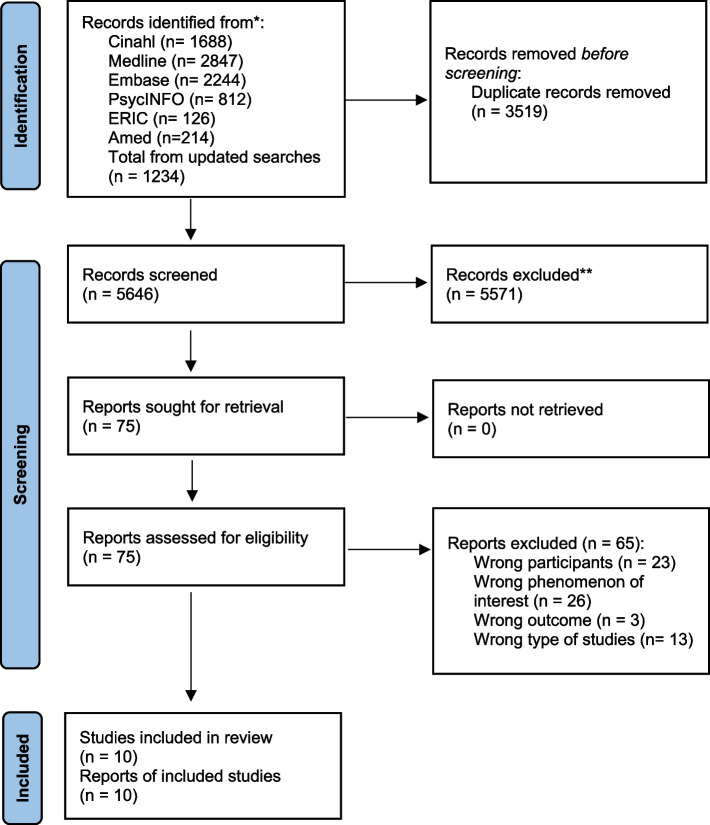
PRISMA 2020 flow diagram

The database searches identified 9165 publications, and the titles and abstracts from 5646 publications were screened after removal of 3519 duplicates. The full text of 75 publications were read, and 10 papers from 10 studies were included. Figure 1 shows the study selection process in the PRISMA 2020 flow diagram [36].

### Description of the studies

The studies included in the review were conducted in the United States (US; *n* = 4), Japan (*n* = 2), the United Kingdom (UK; *n* = 1), Australia (*n* = 1), Sweden (*n* = 1) and Taiwan (*n* = 1). The sample size of the studies ranged from 12 to 160 participants. In nine papers the sample consisted of under 70 participants, while four studies had 20 participants or less. Papers included nurses with varying backgrounds who participated in different education programmes. The participants were advanced practice registered nurse students [37]; family nurse practitioner students [38]; advanced practice registered nurses [39]; graduate nursing students in critical care [40]; postgraduate students in intensive care [41]; acute nurse practitioner students [42]; and nurses employed in palliative care units / inpatient hospice, palliative care consultation teams, general medical wards or medical centres [43–45]. In one paper, participants’ workplaces and specialties were not reported [46].

Two papers used a mixed-methods design [39, 40], three used a pre- and post-test design [38, 42, 44], one used a randomized controlled trial design [43], one was a pilot feasibility study [45], one used a qualitative phenomenographic approach [41], one used a longitudinal survey design [46] and one used an evaluation study design [37].

In three papers the main focus was on communication [42, 44, 45], while two papers focused primarily on communication about advance care planning [38, 46]. Six papers used role playing as the SBL activity [38, 39, 43–46], while six described simulation training with the use of scenarios [37, 38, 40–42, 45]. One study used an online case module first, later combined with a workshop [37]. All the studies described a variety of active participation from the students. The papers described and used different models and tools, including the COMFORT communication model (a holistic, patient-centred communication model consisting of seven tenets [42, 44]), the EXCELL programme (an educational innovation to develop nurses’ intercultural communication skills [40]), the E-FIELD programme (an EOL communication skills training programme [46]), the PREPARED model (a communication guide with eight steps [45]) and the Spiritual Pain Assessment Sheet (a structured assessment sheet to document spiritual pain [43]). The characteristics of the studies included in the review are described in Table 2.

**Table 2:** Characteristics of the included studies

Author, year, country	Aim	Setting and participants	Simulation procedures	Scenarios	Design	Key results
J-O. Chen et al. (2021) Taiwan [45]	To develop and pilot test an advance care planning simulation based communication training program and to evaluate the feasibility and acceptability of the program	Medical center 12 nurses; 12 females, mean age 44.6 (range 26–55) years	Structured clinical communication through scenario simulation, roleplay, and guidance of the PREPARED^a^ model; use of standardized patients, debriefing, reflection and evaluation. Each participant took turn and roleplayed with standardized patients Self-reflection afterwards and feedback given by the facilitator and other members in the group. Timeframe: 25–30 min	Topics for role-play/simulation scenarios: refusing life sustaining treatments, signing documents for advance directive, weaning life sustaining treatment; palliative and home care	Pilot feasibility study	Role-playing was helpful for discovering their own shortcomings in communication skills while debriefing enabled them to identify their blind spots in the communication process. Role-play provided opportunities for peer observation and learning
Ellis et al. (2021) US [38]	To determine the effects of an advance care planning (ACP) communication education module on learner satisfaction and confidence in holding ACP-conversations	Family nurse practitioner graduate nursing program in university 44 students; 40 females; mean age 32.8 (standard deviation (SD) 8.36, range 23–53) years	*First part* was lecture and roleplaying activities where participants took turns playing the patient and the family practice nurse. Timeframe role-play was 1015 min. Debrief afterwards after students had played both roles*. The second* part was clinical simulation 4 weeks later with a standardized patient. Scenario focused on ACP conversations. Students had 15 min for the patient encounter, then time for documenting the conversation. Each simulation experience was followed by a debriefing session with the students, standardized patients and the investigator	*First part of the program (roleplay*): “Breaking Bad News: Case Study # 3” (not reported (NR)) *Second part of the program (simulation):* a son waiting after his mother was emergently taken to hospital with hemorrhagic stroke, loss of consciousness and poor prognosis of survival. The student was the sons first source of information, and the student should break the bad news using the SPIKES^b^ protocol	Pre-test post-test design	Significant increase in post intervention ACP communication knowledge scores (medium effect size), self-confidence in holding these conversations, and satisfaction with the learning experience
Ellman et al. (2012) US [37]	To describe the development, implementation, and evaluation of an innovative program that blends online learning with interactive simulation to teach interprofessional aspects of palliative care (PC)	Interprofessional PC education program Free-text responses: 43 advanced practice registered nurse (APRN) students; 39 females, age NR Postworkshop questionnaire: 65 nursing students, sex NR, age NR	*First part of the program;* online case, embedded video with professional actors addressing goals of care, symptom management, spiritual challenges and family conflict. Timeframe: 30–45. Students enter free-text responses to four reflection questions 90-min interprofessional workshop in groups of 6–8 students and a faculty facilitator: first the group discuss one PC simulation, then 20 min simulation of an interprofessional team meeting. Groups present a summary of their discussions	*First part of the program (online case):* 68-year-old African American woman with end-stage metastatic breast cancer. The case explores clinical challenges in the physical and psychosocial domains with particular emphasis on spiritual and cultural issues *Second part of the program:* a woman of Muslim faith with young children and carcinomatosis with bowel obstruction. With complexity in multiple domains, the case prompts students to experience firsthand the value of interprofessional collaboration	Evaluation study	APRN students experienced the program meaningful, effective and recognized important issues beyond their own discipline, roles of other professionals and the value of teamwork. APRN students experienced the online case and materials as instructive, relevant and challenging, the workshop and discussion groups as valuable and the online interactive case in combination with interdisciplinary discussion groups facilitated learning
Fuoto et al. (2019) US [44]	To increase nurse confidence and satisfaction engaging in end-of-life (EOL) communication following the implementation of the COMFORT^c^ model	PC unit in large tertiary care center 14 nurses; 14 females, mean age 46.9 (SD 11.9) years	4 h COMFORT class; lectures, group discussions, role-play scenarios to demonstrate skills learned. Each participant played the role as patient, nurse and family. After each role-play a debriefing session to discuss lessoned learned and reinforce how to use COMFORT to guide EOL communication	NR	Pre-test post-test design	Significant increase in nurses’ overall ability to communicate in difficult conversations across the 3 time points (before, right after and 3 months after) and a significant increase in satisfaction with managing emotional needs at EOL, managing conflict and overall communication in difficult EOL situations
Gentry and Dahlin (2020) UK [39]	To prove a brief highlight of educational strategies/learn er needs to consider in PC APRN education, describe outcomes of the PC APRN externship in 1 site, and discuss the application of such training in addressing PC workforce needs	A teaching hospital 20 nurse practitioners; sex NR, age NR	A day of classroom training based on the established ELNEC^d^ curriculum with focus on communication. Role-play used as an active learning strategy	Simulated family-meetings, delivering difficult news and eliciting goals of care	Mixed methods design	Most participants reported time spent in clinical areas to be the highlight of the week. Participants spoke of staff enthusiasm, role modeling teamwork contributing to the experience and wanted more practical content about pain and symptom management. Many comments reflected need for validation and support from others who have shared understanding. Participants found active learning strategies helpful
Lindberg et al. (2021) Sweden [41]	To explore postgraduate nursing students´ experiences with simulation training in EOL communication with intensive care patients and their families	Postgraduate diploma studies in Specialist Nursing in Intensive care at university 29 students; 19 females, 4 students gender NR, mean age 35 (range 25–47) years 29 answered a questionnaire, 9 participated in interviews; 6 females	3 h simulation training in groups of 4–6 students. The simulation training took place when students where halfway through intensive care education, in the midst of a 10-week clinical placement in an intensive care unit (ICU). All students were assigned roles as ICU nurse, family member or observer. Reflective seminar after each scenario	*Scenario 1*: Old man with multiple organ failure, been in ICU for several weeks. Decided that life-sustaining treatment will end. Wife and son/daughter part of the scenario *Scenario 2:* 58-year-old woman with severe chronic obstructive lung disease diagnosed several years ago. Treated with CPAP^e^ in ICU without any signs of improvement. Family was informed that she will be moved to an ordinary hospital ward. Husband and son/daughter part of the scenario	Qualitative study with phenomenographic approach	EOL conversations was perceived as being different from the students’ previous experiences. The vulnerability of patients and family members became apparent to the students. The reflections following the simulation scenarios contributed to further development of thoughts about EOL conversations. Scenarios should be as authentic as possible, and information and preparations are important. Uncertainty can hinder learning Students became aware of the importance of being open and reflective when encountering existential issues
Morita et al. (2014) Japan [43]	To determine the impact on nurses of a novel two-day education program focusing on care that addresses patients´ feelings of meaninglessness	Nurses in PC units/inpatient hospices, in PC consultation teams, or on general medical wards Intervention group (*n* = 36); 35 females, age NR Control group (n 40); 39 females, age NR	Two-day educational workshop: lecture, demonstration, role-play and group work based on case vignette using the SpiPas^f^. Nurse facilitators coordinated all the activities After being introduced to the case vignette participants were divided into small groups (6–7 nurses per group) and discussed relevant questions. On day two, each group created nursing care plans through group work and shared them for discussion. Role-play for communication with the patient were then performed	*Case vignette:* Terminally ill cancer patient suffering from increased dependency, being a burden and feeling of meaninglessness	Randomized controlled trial	A significant increase in nurse-reported confidence after the intervention (effect sizes of 0.8). No significant intervention effects identified in self-reported practice, in attitudes toward caring for patients who experience feelings of meaninglessness, in the burnout score, nurses’ own spiritual wellbeing, and knowledge
Northam et al. (2015) Australia [40]	To describe and evaluate the effectiveness of an educational innovation designed to develop graduate-level critical care nurses’ capacity for effective interpersonal communication, as members of a multidisciplinary team in providing culturally sensitive EOL care	Graduate critical care nursing course at university 12 graduate nursing students in critical care, sex NR, Age NR, 5 had and an international background	Weekly workshop (12 workshops): Teaching and learning sessions with integrated and aligned intervention utilizing the EXCELL^g^ tools, vignettes with discussion, simulation, and cases Each student offered opportunity to share a story of their experience of their own culture and experience of death which was used as a discussion prompt and opened a new understanding between the students. Students were encouraged to develop a set of support strategies/a toolbox which were collaboratively developed using their own experienced scenarios and tested in a safe environment in the workshops. In the scenarios students were encouraged to explore the support available for them in complex scenarios, as part of alliance building within the team. A range of vignettes were developed to trigger discussion around EOL care	Care of critically ill child, interprofessional team and actor as mother who are present at the resuscitation bed in the emergency department; complicated cardiogenic shock; new admission to ICU of a critically ill patient when death is probable; interaction with family members with very limited ability to communicate in English language; care of the deceased in the emergency department; when clinical signs indicate the patient is brain dead; when there are indications that withdrawal of life sustaining treatment may be in the patients best interest; communication with families of suicide victims and survivors	A pilot mixed method design	Increase in students’ cultural learning scores in a range of areas including understandings of cultural diversity, interpersonal skills, cross cultural interactions and participating in multicultural groups (no statistical analyses provided). Enhanced levels of nurse confidence in approaching EOL care in both emergency department and intensive care environments
Okada et al. (2021) Japan [46]	To investigate how healthcare providers who attended the E-FIELD^h^ program changed before and after the course in terms of their ACP knowledge, confidence, communication difficulty, and practices	Hospitals with more than 500 beds, hospitals with less than 500 beds and others 160 nurses; 153 females, mean age 43.8 (SD 8.0) years	Ice-breaker, 4-h text-based lecture using a textbook common to all professions, 4 h of small group discussions on a case requiring decision support, 4 h of role-play in ACP-specific scenarios, and discussions on these role-plays. In the role-plays family members were placed in addition to the patient role. Discussions and role-play were conducted in groups of four, consisting of different professions. One facilitator assigned to each group. Facilitators participated in a 10-h training workshop to facilitate communication skills	Scenarios NR in detail, but described as specific to ACP, such as introduction to ACP, selection of surrogate decision maker and decision-making regarding life-sustaining treatment. Family treated as secondpart, considering the cultural characteristics of Japan	Longitudinal survey	Significant increase in knowledge and confidence scores from before training to immediately after training. There was a significant decrease in communication difficulty 6 months after training compared with before training
Roth et al., (2017) US [42]	To evaluate the effectiveness and usefulness of a COMFORT model in strengthening perceived communication confidence of APRN	An acute nurse practitioner program at university 24 adult gerontology acute care nurse practitioner students; 85% females, mean age 37.5 (SD 7.4, age range 27 and 53) years	Simulations completed over a 3 week period during course semester combing a didactic portion of the COMFORT model with a standardized family simulation experience. Participants were introduced to a disorienting dilemma to practice the principles of the COMFORT-model. Debriefing session following the simulation experience	Patient with chronic obstructive pulmonary disease, ischemic heart disease, hypertension and implanted cardiac defibrillator brought into the emergency room with severe respiratory distress. The patient requiring intubation and transfer to ICU. Patient´s exspouse and patient´s sister arguing at odds about the goals of care	Descriptive pre and post survey	Improvements in perceived confidence for initiating potentially difficult communication topics and managing emotional needs of families. APRN positively indicated the COMFORT model as a useful tool for guiding difficult discussion and an effective strategy for guiding APRN communication at EOL Decrease in confidence levels after a SBL session with focus on a difficult family situation, feeling unprepared for family questions, overwhelmed by family emotions, and that they struggled with their role responsibility

^a^ *PREPARED* Prepare for the discussion, Relate to the person, Elicit patient and caregiver preferences, Provide information, Acknowledge emotions and concerns, Encourage questions and further discussions, and Document

^b^ *SPIKES* Setting, Perception, Invitation, Knowledge, Empathy, Summary

^c^ *COMFORT* Communication, Orientation and opportunity, Mindfulness, Family, Openings and oversight, Reiterative and radically adaptive, Team

^d^ *ELNEC* End of Life Nurses Education Consortium

^e^ *CPAP* Continuous Positive Airway Pressure

^f^ *SpiPas* Spiritual Pain Assessment Sheet

^g^ *EXCELL* Excellence in Cultural Experiential Learning and Leadership

^h^ *E-FIELD*: Education for Implementing End of Life Discussion

Three thematic groupings were identified in the data analysis: (1) enhanced understanding of the importance of teamwork, interdisciplinarity and interpersonal skills; (2) preparedness and confidence in one’s ability to communicate during emotionally challenging situations; and (3) impact and relevance to one’s own clinical practice. Table 3 shows the papers included in the thematic groupings.

**Table 3:** Papers included in the thematic groupings

Theme	Study	No. of papers
Enhanced understanding of the importance of teamwork, interdisciplinarity and interpersonal skills	Ellman et al. [37]; Northam et al. [40]; Roth et al. [42]	3
Preparedness and confidence with one’s ability to communicate during emotionally challenging situations	Chen et al. [45]; Ellis et al. [38]; Fuoto et al. [44]; Morita et al. [43]; Okada et al. [46]; Roth et al. [42]	6
Impact and relevance to one’s own clinical practice	Chen et al. [45]; Fuoto et al. [44]; Gentry et al. [39]; Lindberg et al. [41]; Roth et al. [42]	5

### Thematic groupings

#### Enhanced understanding of the importance of teamwork, interdisciplinarity and interpersonal skills

In two of the studies, the participants highlighted how SBL had enhanced their understanding of the importance of teamwork and interdisciplinarity in palliative care [37, 42]. After participating in SBL, participants also reported that they had a greater understanding of the contributions of other healthcare professionals and the importance of the interdisciplinary team in the care of dying patients [37]. Common themes in several debriefings included reflecting and discussing the importance of interdisciplinary approaches, coordinating family meetings and engaging other disciplines when faced with families in crisis situations [42]. In one study that focused on participation in multicultural groups, the participants felt the training to be useful, and they were more confident and comfortable after participating in the SBL [40].

#### Preparedness and confidence in one’s ability to communicate during emotionally challenging situations

The participants reported that SBL had improved their ability to communicate during difficult conversations [42, 44, 45]. Participants felt a significant increase in satisfaction and competence in their management of emotional needs at the EOL [44]. The use of SBL improved the participants’ ability to initiate potentially difficult communication topics and to manage the emotional needs of patients and families, and it increased their overall confidence in communicating during difficult situations [42]. Another study noted a significant increase in the participants’ competence in communicating with families in crisis [44]. After participants were involved in a two-day workshop with a focus on care that addressed patients’ feelings of meaninglessness, there was a significant positive effect observed in the participants reported confidence after the intervention [43]. Participants in two different educational programmes with a focus on advance care planning in SBL showed a significant increase in advance care planning communication knowledge and self-confidence in holding these conversations [38, 46].

In one study, the participants had to manage a conflict among family members concerning the goals of care [42]. The participants positively evaluated the COMFORT model as an effective strategy for guiding difficult discussions. The participants experienced a decrease in confidence levels after an SBL session with a focus on a difficult family situation, however. During the debriefing, these students expressed that they felt unprepared for the family’s questions and felt overwhelmed by the family’s emotions, and they struggled with the responsibility of their role [42].

#### Impact and relevance to one’s own clinical practice

In three of the studies, participants indicated improved communication in their practice as nurses following the use of SBL [39, 44, 45]. For example, participants highlighted that they felt more confident, prepared and aware after the COMFORT training [44] and that they could use the COMFORT model as a guide for communication in their daily practice [42]. In another study, participants reported that they were more patient when communicating with patients after the training, and they tried to explore the real meaning of what the patients were saying [45]. Participants wanted more practical content during SBL, however, with a focus on pain and symptom management [39].

Regarding the application to practice, the participants’ responses consisted of specific changes to future clinical practice. Some participants indicated improved communication and symptom-management skills, while others spoke of programme-development ideas. On a more personal note, participants spoke of personal growth and development [39]. Others reflected on the use of the COMFORT model as a useful guide for effective communication and a reminder of the importance of a team approach. Other participants offered insight to future practice that would incorporate listening more deeply and addressing the grief, guilt and anticipatory loss of families who face difficult EOL decision-making [42]. In one study, participants pointed out that acting as a relative in the scenario gave them new insights from a new perspective into the family members’ experience of vulnerability during the situation [41]. The participants saw reflection after the SBL as useful for their own clinical practice in various ways as they became better acquainted with their weaknesses [45] and experienced courage and strength to meet patients and families in clinical practice [41].

## Discussion

The aim of this scoping review was to systematically map published studies on the use of SBL in palliative care in postgraduate nursing education. Our findings indicate that SBL enhanced postgraduate nursing students’ understanding of the importance of teamwork and interdisciplinarity in palliative care. The students also gained an enhanced ability to communicate during difficult conversations after participating in SBL, and they experienced personal growth and development after participation in SBL. In one study, however, the postgraduate students reported decreased confidence in their communication with families [42].

Interprofessional teamwork is essential when delivering high-quality palliative care [47]. The participants in Klarare et al.’s [48] study had long experience of working in palliative care teams but emphasized that teamwork was challenging, as was working interprofessionally and not in a multidisciplinary manner. Multidisciplinary teams consist of different professionals working in parallel, while interprofessional teams work together, share information and are involved in making joint decisions based on a patient-centred approach [49]. The World Health Organization (WHO) [50] recommends interprofessional activity in education to promote collaboration in healthcare and underlines that the only way healthcare professionals can understand and be ready to collaborate interprofessionally is through interprofessional focus and training in education. SBL may be used to enhance the interprofessional understanding of roles and enable interprofessional teamwork [51].

We were able to include only ten studies, indicating that this is a limited research field. Of the ten studies included in our scoping review, six primarily focused on communication training in different forms. SBL is generally recommended as a learning activity to teach communication skills in palliative care [14] and has been used to develop skills related to communication and the provision of EOL care [52]. Communication is one of several important competencies for nurses in palliative care [9, 53, 54], and the most common form of SBL in palliative care is role playing with an actor and a focus on communication skills [55]. Healthcare professionals may feel anxious and unprepared to talk about death and EOL [56]. Nurses in palliative care may perceive communication with families as challenging, since they may lack competence within this area of communication [1]. Our findings suggest that even though the use of SBL enhanced postgraduate nursing students’ confidence in their communication skills, in one of the studies [42] in our review, the students experienced decreased confidence levels after an SBL session that focused on a difficult family situation. The nursing students felt overwhelmed and unprepared, and they struggled with role responsibility [42]. Students may feel frustrated after participating in SBL, and they may need constructive feedback and a skilled educator to help them transform feelings of mistakes into a positive learning experience [57].

Novaes et al. [52] concluded in their study that the facilitator is the most crucial factor in the implementation of role playing for teaching communication skills in palliative care. Effective facilitation of an SBL experience requires a facilitator who has specific skills and knowledge in simulation pedagogy, and the facilitator’s competence can be key for the participants’ opportunities to learn and to be able to achieve the expected learning outcomes [58]. Two of the studies included in our review did not report whether a facilitator was present [39, 44], and no studies described facilitator characteristics such as experience, training, profession or sex, as recommended by Cheng et al. [59]. Sevdalis et al. [60] found a lack of consistent reporting and the potential for improvement in the quality of reporting on SBL research.

Palliative care patients are especially vulnerable [61], and the provision of palliative care can be stressful because of the emotional impact caused by the frequent contact with suffering and death [62, 63]. Palliative care nurses must have personal resources to cope with stressful events, and personal growth is often considered an important protective factor [64]. Our findings indicate that postgraduate nursing students felt more confident after participating in SBL and experienced personal growth and development, but some of the participants wanted more practical content, such as symptom management. Postgraduate nursing students should receive relevant training and education to feel prepared for meetings with patients and their families during palliative care. Inadequate skills, capacities, education and training for nurses could be potential barriers to palliative care [14, 65].

Various reasons could explain the low number of studies included in our review. Since several countries do not offer postgraduate education in palliative care nursing [8], nurses could be offered shorter courses and receive training related to palliative care that includes SBL at their workplaces [66–68]. Providing education in palliative care may also pose challenges due to possible lack of faculty expertise [54]. Palliative medicine is also a relatively young specialty [23], and education in the palliative field has been slow to use SBL [55]. One reason for this slow introduction of SBL into the palliative field may be due to lack of financial resources as SBL could be an expensive and time-consuming learning method [55]. There is a focus on required competencies in palliative care for nurses such as communication, observation, evaluation, symptom management and collaboration [53, 54], and SBL in addition to other active learning methods are recommended in undergraduate and postgraduate education [14, 69]. Gillan et al. [28] found that SBL with a focus on teaching undergraduate nursing students EOL skills was first used in 2009. SBL may be perceived as less relevant in palliative care because such care is often considered a less acute specialty than, for example, anaesthetics [55]. High-technology simulation for learning and teaching for example resuscitation has experienced comprehensive growth [70]. Palliative care may be described as ‘high touch’ [71], and André et al. [72] have noted a conflict between high tech and high touch in the palliative field. SBL can be low tech, however, and still maintain high fidelity [73].

Future research should explore postgraduate nursing students’ experiences with symptom management using standardized patients and manikin-based or hybrid simulations and determine whether such simulation strategies can improve the students’ symptom-management skills. Based on the crucial role of the facilitator, the field requires research with thorough descriptions of how simulations are carried out, what the facilitator’s role is, and the facilitator’s background and education. The scenarios should be described in a more in-depth manner, in line with guidelines on reporting on SBL research. Research focused on how participants experience the relevance and application of SBL within their own clinical practice is also lacking at the postgraduate level.

## Strengths and limitations

One strength of this review was the use of an acknowledged framework for conducting scoping reviews, as described by Arksey and O’Malley [30], while the reporting was supported by the PRISMA-ScR checklist [31]. The development of the search strategy and the comprehensive search for published studies was done in close cooperation with an experienced research librarian, and the search strategy was discussed several times and peer reviewed by another research librarian. The study selection and data extraction were done individually in pairs. Our protocol was published before the database searches, and study selection was performed.

We excluded studies where postgraduate nursing students participated in SBL with different disciplines and where the studies did not report separate results for postgraduate nursing students. Because of language limitations, we may have excluded some relevant studies. The studies included in this scoping review featured a variety of types, scopes and duration of SBL, which may have affected the findings. Because we did not assess the methodological quality of the studies nor synthesise their findings, as recommended in the literature [30], any implications for education and policy should be interpreted with caution.

## Conclusion

The use of simulation-based learning (SBL) in postgraduate nursing education seems to enhance students’ understanding of the importance of teamwork and interdisciplinarity, which is a challenging, but crucial, part of providing palliative care. Even though most of our evidence suggests that SBL enhanced postgraduate nursing students’ confidence in their communication skills, we also found contradictory findings. The students experienced personal growth after participating in SBL, which can be an important protective factor against stress. Our findings suggest that limited research has been conducted within this field and that facilitator characteristics and scenarios have been poorly reported. Future research should explore postgraduate nursing students’ experiences with SBL, with a focus on more practical content and other essential competencies within palliative care such as clinical and ethical competencies and on SBL’s relevance and application within the nurses’ own clinical practice. Future research should also be reported in line with recommendations regarding the reporting of SBL research.

## Supplementary Information

**Additional file 1: Appendix 1.** Deviations from the protocol.

**Additional file 2: Appendix 2.** Search histories.

## Data Availability

All data generated or analysed during this study are included in this published article and its supplementary information files.

## Abbreviations

ACP: Advance care planning
AMED: Allied and Complementary Medicine
APRN: Advanced practice registered nurses
CINAHL: Cumulative Index to Nursing and Allied Health Literature
CPAP: Continuous positive airway pressure
EOL: End-of-life
ERIC: Education Resources Information
ICU: Intensive care unit
NR: Not reported
PC: Palliative care
PRESS: Peer Review of Electronic Search Strategies
PRISMA: Preferred Reporting Items for Systematic Reviews and Meta-Analyses
SBL: Simulation-based learning
ScR: Scoping review
SD: Standard deviation
UK: United Kingdom
US: United States
WHO: World Health Organization
