# Supplementary material for: Simulation-based learning in palliative care in postgraduate nursing education: a scoping review
Source: BMC Palliat Care. 2023 Mar 29;22:30. doi: 10.1186/s12904-023-01149-w (PMC10052798; doi:10.1186/s12904-023-01149-w)
Supplement: Supplementary file 1 — Additional file 1: Appendix 1. Deviations from the protocol. [file 12904_2023_1149_MOESM1_ESM.pdf]

Appendix 1 Deviations from the protocol

**Inclusion criteria described in the protocol**	**Description and justification of the deviation from the protocol**
Nursing students in postgraduate education, regardless of type of education and course, duration and extent of course/education	We included papers where nurses participated in SBL in clinical practice when SBL was based on comprehensive activity such as curricula, models, textbooks or other tools; we found these papers relevant for the research question, and such papers can provide insight into postgraduate nurses' experiences with SBL
