# Supplementary material for: Simulation-based learning in palliative care in postgraduate nursing education: a scoping review
Source: BMC Palliat Care. 2023 Mar 29;22:30. doi: 10.1186/s12904-023-01149-w (PMC10052798; doi:10.1186/s12904-023-01149-w)
Supplement: Supplementary file 2 — Additional file 2: Appendix 2. Search histories. [file 12904_2023_1149_MOESM2_ESM.pdf]

Appendix 2 Search histories
All searches last run 21st of April 2022

Cinahl via Ebsco Host

#	Query	Limiters/Expanders
S51	S22 AND S48	Limiters - Published Date: 20000101-20211231; Language: Danish, Dutch/Flemish, English, French, Norwegian, Portuguese, Spanish, Swedish<br>Search modes - Boolean/Phrase
S50	S22 AND S48	Limiters - Published Date: 20000101-20211231<br>Search modes - Boolean/Phrase
S49	S22 AND S48	Search modes - Boolean/Phrase
S48	S23 OR S24 OR S25 OR S26 OR S27 OR S28 OR S29 OR S30 OR S31 OR S32 OR S33 OR S34 OR S35 OR S36 OR S37 OR S38 OR S39 OR S40 OR S41 OR S42 OR S43 OR S44 OR S45 OR S46 OR S47	Search modes - Boolean/Phrase
S47	TI (Programmed Instruction) OR AB (Programmed Instruction)	Search modes - Boolean/Phrase
S46	TI ((computer OR multimedia) N4 (program* OR education* OR environment* OR learn* OR interface* OR app OR application* OR instruction*)) OR AB ((computer OR multimedia) N4 (program* OR education* OR environment* OR learn* OR interface* OR app OR application* OR instruction*))	Search modes - Boolean/Phrase
S45	TI (game* OR gaming) OR AB (game* OR gaming)	Search modes - Boolean/Phrase
S44	TI ((Practical OR practice) n1 (Lab or labs or laborator*)) OR AB ((Practical OR practice) n1 (Lab or labs or laborator*))	Search modes - Boolean/Phrase
S43	TI (Learning n1 (Lab or labs or laborator*)) OR AB (Learning n1 (Lab or labs or laborator*))	Search modes - Boolean/Phrase
S42	TI (skill* n1 (Lab or labs or laborator*)) OR AB (skill* n1 (Lab or labs or laborator*))	Search modes - Boolean/Phrase
S41	TI ((Role* n0 play*) OR roleplay*) OR AB ((Role* n0 play*) OR roleplay*)	Search modes - Boolean/Phrase
S40	TI (Anatomic n1 Model*) OR AB (Anatomic n1 Model*)	Search modes - Boolean/Phrase

S39	TI (Standardi#ed n1 Patient*) OR AB (Standardi#ed n1 Patient*)	Search modes - Boolean/Phrase
S38	TI (skill* N0 training*) OR AB (skill* N0 training*)	Search modes - Boolean/Phrase
S37	TI ("high-fidelity" or "low-fidelity") OR AB ("high-fidelity" or "low-fidelity")	Search modes - Boolean/Phrase
S36	TI (manikin* or mannequin*) OR AB (manikin* or mannequin*)	Search modes - Boolean/Phrase
S35	TI (simulat* OR SBL) OR AB (simulat* OR SBL)	Search modes - Boolean/Phrase
S34	TI ((virtual OR augmented) n1 realit*)) OR AB ((virtual OR augmented) n1 realit*))	Search modes - Boolean/Phrase
S33	TI "VR" OR AB "VR"	Search modes - Boolean/Phrase
S32	(MH "Virtual Reality+")	Search modes - Boolean/Phrase
S31	(MH "Augmented Reality")	Search modes - Boolean/Phrase
S30	(MH "Learning Environment, Clinical")	Search modes - Boolean/Phrase
S29	(MH "Learning Laboratories")	Search modes - Boolean/Phrase
S28	(MH "Games+")	Search modes - Boolean/Phrase
S27	(MH "Role Playing")	Search modes - Boolean/Phrase
S26	(MH "Programmed Instruction+")	Search modes - Boolean/Phrase
S25	(MH "Computer Assisted Instruction")	Search modes - Boolean/Phrase
S24	(MH "Models, Anatomic+")	Search modes - Boolean/Phrase
S23	(MH "Simulations+")	Search modes - Boolean/Phrase
S22	S1 OR S2 OR S3 OR S4 OR S5 OR S6 OR S7 OR S8 OR S9 OR S10 OR S11 OR S12 OR S13 OR S14 OR S15 OR S16 OR S17 OR S18 OR S19 OR S20 OR S21	Search modes - Boolean/Phrase
S21	TI (DNR or DNI) OR AB (DNR or DNI)	Search modes - Boolean/Phrase
S20	TI ("do not" N0 (intubate or resuscitate)) OR AB ("do not" N0 (intubate or resuscitate))	Search modes - Boolean/Phrase
S19	TI (advance* N2 (plan or plans or planning or directive*)) OR AB (advance* N2 (plan or plans or planning or directive*))	Search modes - Boolean/Phrase
S18	TI ((dying or (right N1 die) or (die N1 dignity)) OR AB ((dying or (right N1 die) or (die N1 dignity))	Search modes - Boolean/Phrase
S17	TI (supporti* n1 care) OR AB (supporti* n1 care)	Search modes - Boolean/Phrase
S16	TI "comfort measure*" OR AB "comfort measure*"	Search modes - Boolean/Phrase
S15	(MH "Hospices")	Search modes - Boolean/Phrase

S14	(MH "Hospice Patients")	Search modes - Boolean/Phrase
S13	TI hospice* OR AB hospice*	Search modes - Boolean/Phrase
S12	TI (("life limit*" or "life threat*") N1 (illness* or disease* or condition* or cancer*)) OR AB (("life limiti*" or "life threat*") N1 (illness* or disease* or condition* or cancer*))	Search modes - Boolean/Phrase
S11	TI ((terminal* or "end-stage*" or "advanced-stage*" or "late-stage*") N2 (disease* or condition* or sympt* or ill* or care* or caring* or treatment* or period* or nurs* or patient*)) OR AB ((terminal* or "end-stage*" or "advanced-stage*" or "late-stage*") N2 (disease* or condition* or sympt* or ill* or care* or caring* or treatment* or period* or nurs* or patient*))	Search modes - Boolean/Phrase
S10	TI (palliative or palliate* or palliating) OR AB (palliative or palliate* or palliating)	Search modes - Boolean/Phrase
S9	TI (eol or "end of life") OR AB (eol or "end of life")	Search modes - Boolean/Phrase
S8	(MH "Right to Die")	Search modes - Boolean/Phrase
S7	(MH "Resuscitation Orders")	Search modes - Boolean/Phrase
S6	(MH "Advance Care Planning")	Search modes - Boolean/Phrase
S5	(MH "Terminally Ill Patients+")	Search modes - Boolean/Phrase
S4	(MH "Hospice Care")	Search modes - Boolean/Phrase
S3	(MH "Terminal Care")	Search modes - Boolean/Phrase
S2	(MH "Hospice and Palliative Nursing")	Search modes - Boolean/Phrase
S1	(MH "Palliative Care")	Search modes - Boolean/Phrase

Ovid MEDLINE(R) and Epub Ahead of Print, In-Process & Other Non-Indexed Citations and Daily

#	Searches
1	Palliative Care/
2	Hospices/
3	"Hospice and Palliative Care Nursing"/
4	Hospice Care/
5	Terminal Care/
6	Palliative Medicine/
7	exp Advance Care Planning/
8	Resuscitation Orders/
9	"Right to Die"/
10	Terminally ill/
11	(palliative or palliate* or palliating).tw,kf.
12	(dying or (right adj2 die) or (die adj2 dignity)).tw,kf.
13	(supporti* adj2 care).tw,kf.
14	((terminal* or "end stage*" or "advanced stage*" or "late stage*") adj3 (disease* or ill* or care* or caring or treatment* or period* or nurs* or patient*)).tw,kf.
15	(eol or "end of life").tw,kf.
16	(("life limiting" or "life threatening") adj3 (disease* or condition* or illness*)).tw,kf.
17	("do not" adj1 (intubate or resuscitate)).tw,kf.
18	(DNR or DNI).tw,kf.
19	"comfort measure*".tw,kf.
20	(advance*1 adj3 (plan*1 or planning or directive*)).tw,kf.
21	hospice*.tw,kf.
22	or/1-21
23	Computer Simulation/
24	augmented reality/
25	virtual reality/
26	Patient-Specific Modeling/
27	exp Simulation Training/
28	Models, Anatomic/
29	Manikins/
30	exp Programmed Instructions as Topic/
31	Role Playing/
32	Video Games/
33	vr.tw,kf.
34	((virtual* or augmented) adj2 realit*).tw,kf.
35	simulat*.tw,kf.
36	sbl.tw,kf.

37	(manikin* or mannequin*).tw,kf.
38	("high-fidelity" or "low-fidelity").tw,kf.
39	(skill* adj2 training*).tw,kf.
40	(Standardi#ed adj2 Patient*).tw,kf.
41	(Anatomic adj2 Model*).tw,kf.
42	(Role* adj1 play*).tw,kf.
43	(skill* adj (Lab or labs or laborator*)).tw,kf.
44	(Learning adj2 (Lab or labs or laborator*)).tw,kf.
45	((Practical or practice) adj (Lab or labs or laborator*)).tw,kf.
46	(game* or gaming).tw,kf.
47	((computer or multimedia) adj5 (program* or education* or environment* or learn* or interface* or app or application* or instruction*)).tw,kf.
48	Programmed Instruction.tw,kf.
49	or/23-48
50	22 and 49
51	limit 50 to (yr="2000 -Current" and (danish or dutch or english or flemish or french or norwegian or portuguese or spanish or swedish))
52	limit 51 to (comment or editorial)
53	51 not 52
54	remove duplicates from 53

Ovid Embase

#	Searches
1	exp palliative therapy/
2	palliative nursing/
3	hospice/
4	terminal care/
5	advance care planning/
6	hospice care/
7	hospice nursing/
8	right to die/
9	exp terminally ill patient/
10	(palliative or palliate* or palliating).tw,kw.
11	(dying or (right adj2 die) or (die adj2 dignity)).tw,kw.
12	(supporti* adj2 care).tw,kw.
13	((terminal* or "end stage*" or "advanced stage*" or "late stage*") adj3 (disease* or ill* or care* or caring or treatment* or period* or nurs* or patient*)).tw,kw.
14	(eol or "end of life").tw,kw.
15	(("life limiting" or "life threatening") adj3 (disease* or condition* or illness*)).tw,kw.
16	("do not" adj1 (intubate or resuscitate)).tw,kw.
17	(DNR or DNI).tw,kw.
18	"comfort measure*".tw,kw.
19	(advance*1 adj3 (plan*1 or planning or directive*)).tw,kw.
20	hospice*.tw,kw.
21	or/1-20
22	simulation/
23	exp computer simulation/
24	exp high-fidelity simulation/
25	exp patient simulation/
26	vignette/
27	simulation training/
28	high fidelity simulation training/
29	simulator/
30	breathing simulator/
31	exp high-fidelity simulator/
32	exp patient simulator/
33	virtual reality simulator/
34	virtual reality head mounted display/
35	exp augmented reality system/
36	virtual reality system/

37	human machine interface/
38	anatomic model/
39	manikin/
40	role playing/
41	video game/
42	vr.tw,kw.
43	((virtual* or augmented) adj2 realit*).tw,kw.
44	simulat*.tw,kw.
45	SBL.tw,kw.
46	(manikin* or mannequin*).tw,kw.
47	("high-fidelity" or "low-fidelity").tw,kw.
48	(skill* adj2 training*).tw,kw.
49	(Standardi#ed adj2 Patient*).tw,kw.
50	(Anatomic adj2 Model*).tw,kw.
51	(Role* adj1 play*).tw,kw.
52	(skill* adj (Lab or labs or laborator*)).tw,kw.
53	(Learning adj2 (Lab or labs or laborator*)).tw,kw.
54	((Practical or practice) adj (Lab or labs or laborator*)).tw,kw.
55	(game* or gaming).tw,kw.
56	((computer or multimedia) adj5 (program* or education* or environment* or learn* or interface* or app or application* or instruction*)).tw,kw.
57	Programmed Instruction.tw,kw.
58	or/22-56
59	21 and 58
60	limit 59 to ((danish or dutch or english or french or norwegian or portuguese or spanish or swedish) and yr="2000 -Current")
61	limit 60 to embase
62	remove duplicates from 61
63	limit 62 to (conference abstract or editorial or letter)
64	62 not 63

Ovid PsycINFO

#	**Searches**
1	palliative care/
2	symptoms based treatment/
3	hospice/
4	terminally ill patients/
5	"Death and dying"/
6	advance directives/
7	(palliative or palliate* or palliating).tw.
8	(dying or (right adj2 die) or (die adj2 dignity)).tw.
9	(supporti* adj2 care).tw.
10	((terminal* or "end stage*" or "advanced stage*" or "late stage*") adj3 (disease* or ill* or care* or caring or treatment* or period* or nurs* or patient*)).tw.
11	(eol or "end of life").tw.
12	(("life limiting" or "life threatening") adj3 (disease* or condition* or illness*)).tw.
13	("do not" adj1 (intubate or resuscitate)).tw.
14	(DNR or DNI).tw.
15	"comfort measure*".tw.
16	(advance*1 adj3 (plan*1 or planning or directive*)).tw.
17	hospice*.tw.
18	or/1-17
19	simulation/
20	computer simulation/
21	simulation games/
22	exp virtual reality/
23	computer games/
24	digital game-based learning/
25	Role Playing Games/
26	computer supported collaborative learning/
27	role playing/
28	role taking/
29	computer assisted instruction/
30	vr.tw.
31	((virtual* or augmented) adj2 realit*).tw.
32	simulat*.tw.
33	SBL.tw.
34	(manikin* or mannequin*).tw.
35	("high-fidelity" or "low-fidelity").tw.
36	(skill* adj2 training*).tw.

37	(Standardi#ed adj2 Patient*).tw.
38	(Anatomic adj2 Model*).tw.
39	(Role* adj1 play*).tw.
40	(skill* adj (Lab or labs or laborator*)).tw.
41	(Learning adj2 (Lab or labs or laborator*)).tw.
42	((Practical or practice) adj (Lab or labs or laborator*)).tw.
43	(game* or gaming).tw.
44	((computer or multimedia) adj5 (program* or education* or environment* or learn* or interface* or app or application* or instruction*)).tw.
45	Programmed Instruction.tw.
46	or/19-45
47	18 and 46
48	remove duplicates from 47
49	limit 48 to yr="2000 -Current"
50	limit 49 to (danish or dutch or english or french or norwegian or portuguese or spanish or swedish)
51	limit 50 to (editorial or letter)
52	50 not 51

ERIC via EBSCO

#	**Query**
S44	S15 AND S41
S43	S15 AND S41
S42	S15 AND S41
S41	S16 OR S17 OR S18 OR S19 OR S20 OR S21 OR S22 OR S23 OR S24 OR S25 OR S26 OR S27 OR S28 OR S29 OR S30 OR S31 OR S32 OR S33 OR S34 OR S35 OR S36 OR S37 OR S38 OR S39 OR S40
S40	TI (Programmed Instruction) OR AB (Programmed Instruction)
S39	TI ((computer OR multimedia) N4 (program* OR education* OR environment* OR learn* OR interface* OR app OR application* OR instruction*)) OR AB ((computer OR multimedia) N4 (program* OR education* OR environment* OR learn* OR interface* OR app OR application* OR instruction*))
S38	TI (game* OR gaming) OR AB (game* OR gaming)
S37	TI ((Practical OR practice) n1 (Lab or labs or laborator*)) OR AB ((Practical OR practice) n1 (Lab or labs or laborator*))
S36	TI (skill* n1 (Lab or labs or laborator*)) OR AB (skill* n1 (Lab or labs or laborator*))
S35	TI (skill* n1 (Lab or labs or laborator*)) OR AB (skill* n1 (Lab or labs or laborator*))
S34	

	TI ((Role* n0 play*) OR roleplay*) OR AB ((Role* n0 play*) OR roleplay*)
S33	TI (Anatomic n1 Model*) OR AB (Anatomic n1 Model*)
S32	TI (Standardi#ed n1 Patient*) OR AB (Standardi#ed n1 Patient*)
S31	TI (skill* N0 training*) OR AB (skill* N0 training*)
S30	TI ("high-fidelity" or "low-fidelity") OR AB ("high-fidelity" or "low-fidelity")
S29	TI (manikin* or mannequin*) OR AB (manikin* or mannequin*)
S28	TI (simulat* OR SBL) OR AB (simulat* OR SBL)
S27	TI ((virtual OR augmented) n1 realit*)) OR AB ((virtual OR augmented) n1 realit*))
S26	TI "VR" OR AB "VR"
S25	DE "Electronic Learning"
S24	DE "Interactive Video"
S23	DE "Computer Mediated Communication")

S22	DE "Computer Assisted Instruction"
S21	DE "Vignettes"
S20	DE "Simulated Environment"
S19	DE "Educational Games"
S18	DE "Role Playing"
S17	DE "Computer Simulation"
S16	DE "Simulation"
S15	S1 OR S2 OR S3 OR S4 OR S5 OR S6 OR S7 OR S8 OR S9 OR S10 OR S11 OR S12 OR S13 OR S14
S14	TI (DNR or DNI) OR AB (DNR or DNI)
S13	TI ("do not" N0 (intubate or resuscitate)) OR AB ("do not" N0 (intubate or resuscitate))
S12	

	TI (advance* N2 (plan or plans or planning or directive*)) OR AB (advance* N2 (plan or plans or planning or directive*))
S11	TI ((dying or (right N1 die) or (die N1 dignity)) OR AB ((dying or (right N1 die) or (die N1 dignity))
S10	TI (supporti* n1 care) OR AB (supporti* n1 care)
S9	TI "comfort measure*" OR AB "comfort measure*"
S8	TI hospice* OR AB hospice*
S7	TI (("life limit*" or "life threat*") N1 (illness* or disease* or condition* or cancer*)) OR AB (("life limiti*" or "life threat*") N1 (illness* or disease* or condition* or cancer*))
S6	TI ((terminal* or "end-stage*" or "advanced-stage*" or "late-stage*") N2 (disease* or condition* or sympt* or ill* or care* or caring* or treatment* or period* or nurs* or patient*)) OR AB ((terminal* or "end-stage*" or "advanced-stage*" or "late-stage*") N2 (disease* or condition* or sympt* or ill* or care* or caring* or treatment* or period* or nurs* or patient*))
S5	TI (palliative or palliate* or palliating) OR AB (palliative or palliate* or palliating)
S4	TI (eol or "end of life") OR AB (eol or "end of life")
S3	DE "Death"
S2	DE "Terminal Illness"

S1	DE "Hospices (Terminal Care)"

Ovid Amed (Allied and complementary medicine)

#	Searches
1	palliative care/
2	exp terminal care/
3	palliative medicine/
4	advance directives/
5	Resuscitation Orders/
6	"Right to Die"/
7	terminal illness/
8	(palliative or palliate* or palliating).tw.
9	(dying or (right adj2 die) or (die adj2 dignity)).tw.
10	(supporti* adj2 care).tw.
11	((terminal* or "end stage*" or "advanced stage*" or "late stage*") adj3 (disease* or ill* or care* or caring or treatment* or period* or nurs* or patient*)).tw.
12	(eol or "end of life").tw.
13	(("life limiting" or "life threatening") adj3 (disease* or condition* or illness*)).tw.
14	("do not" adj1 (intubate or resuscitate)).tw.
15	(DNR or DNI).tw.
16	"comfort measure*".tw.
17	(advance*1 adj3 (plan*1 or planning or directive*)).tw.
18	hospice*.tw.
19	or/1-18
20	computer simulation/
21	computer assisted instruction/
22	virtual reality/
23	role playing/
24	vr.tw.
25	((virtual* or augmented) adj2 realit*).tw.
26	simulat*.tw.
27	SBL.tw.
28	(manikin* or mannequin*).tw.
29	("high-fidelity" or "low-fidelity").tw.
30	(skill* adj2 training*).tw.
31	(Standardi#ed adj2 Patient*).tw.
32	(Anatomic adj2 Model*).tw.
33	(Role* adj1 play*).tw.
34	(skill* adj (Lab or labs or laborator*)).tw.
35	(Learning adj2 (Lab or labs or laborator*)).tw.

36	((Practical or practice) adj (Lab or labs or laborator*)).tw.
37	(game* or gaming).tw.
38	((computer or multimedia) adj5 (program* or education* or environment* or learn* or interface* or app or application* or instruction*)).tw.
39	Programmed Instruction.tw.
40	or/20-39
41	19 and 40
42	limit 41 to yr="2000 -Current"
43	limit 42 to (danish or dutch or english or french or norwegian or portuguese or spanish or swedish)
44	remove duplicates from 43
